# Percutaneous Aponeurotomy and Lipofilling in Complex Periorbital Scar Revision

**DOI:** 10.1002/wjo2.70115

**Published:** 2026-04-29

**Authors:** Daniel Karasik, Alexandra F. Welschmeyer, Eve Tranchito, Humzah Quereshy, Saikrishna Gourishetti, Cyrus C. Rabbani

**Affiliations:** ^1^ Department of Otolaryngology‐Head and Neck Surgery University Hospitals Cleveland Medical Center Cleveland Ohio USA; ^2^ Case Western Reserve University School of Medicine Cleveland Ohio USA

**Keywords:** autologous fat grafting, eyelid retraction, ocular prosthesis, percutaneous aponeurotomy and lipofilling, periorbital scar, scar contracture, scar revision, structural fat grafting

## Abstract

Periorbital scars and deformities pose a significant challenge due to the region's delicate anatomy and esthetic importance. This case series explores the application of percutaneous aponeurotomy and lipofilling (PALF) in three patients with complex periorbital scarring resulting from surgical, traumatic, and pathological causes. The PALF technique involves subcision to release fibrotic tissue tethering scars, followed by injection of processed autologous fat to restore volume and improve contour. Patients demonstrated notable improvements in functional outcomes, such as eyelid mobility and prosthesis positioning, and esthetic results, including enhanced symmetry and scar improvement. No significant complications were observed. This study highlights PALF as a safe and effective treatment for restoring function and esthetics in the management of periorbital scars. Further research is warranted to validate its long‐term efficacy and broader applicability.

## Keypoints

Percutaneous aponeurotomy and lipofilling (PALF) effectively addresses complex periorbital scars, achieving significant functional and esthetic improvements with minimal complications in cases of trauma, surgery, and malignant pathology.

## 1 Introduction

Addressing complex injuries in the periorbital region poses a formidable challenge due to the area's intricate anatomy and delicate structures. Lesions in this region may lead to complications, including asymmetry, volume loss, scarring, and functional impairment. This case series presents three patients who underwent percutaneous aponeurotomy and lipofilling (PALF) within the periorbital region for a range of lesions, including post‐surgical scars and traumatic injury. PALF has previously been described by Khouri et al., although primarily in the setting of scar contractures and soft‐tissue deficiencies throughout the body [1]. Since then, PALF has evolved to treat atrophic acne scars and burn wounds and to manage Dupuytren contracture [2, 3].

Reports describing the application of percutaneous aponeurotomy and structural fat grafting in the periorbital region remain limited [4]. This case series describes the application of PALF for the treatment of periorbital scars and deformities in three patients with significant scarring in this area, resulting in favorable esthetic and functional outcomes. The method involves percutaneous aponeurotomy to release scar tissue, followed by injection of autologous fat, usually harvested from the abdomen [5]. The goal is to restore volume and function, and improve symmetry and esthetic outcomes. Our findings demonstrate the effective application of PALF in each case, not only in restoring functionality but also in enhancing esthetic outcomes for each patient. Following treatment, each patient presented herein demonstrated significant functional and esthetic improvements with minimal adverse effects. While the use of PALF for complex periorbital scar revision is not yet widespread, these cases highlight its potential as a valuable addition to the repertoire of techniques for facial plastic and reconstructive surgeons.

## 2 Methods

### 2.1 Percutaneous Aponeurotomy Procedure

Percutaneous aponeurotomy involves using a cannula to sever subcutaneous fibrotic attachments that tether scar tissue, thereby creating space deep to the scarred area. This enables the overlying tissue to be elevated to the level of the surrounding skin.

### 2.2 Fat Harvesting and Grafting

Liposuction is performed to harvest fat, typically from the patient's abdomen. The harvested fat can then be processed using the Tulip NanoTransfer kit (Tulip Medical Inc., San Diego, CA). Depending on the treatment plane, fat was injected as a structural graft for volume restoration or further mechanically refined for use in more superficial planes to optimize contour and tissue quality.

## 3 Results

### 3.1 Patient 1

Patient 1 presented with a history of midface malignancy and subsequent free‐flap reconstruction, complicated by seroma and infection. He had significant scarring around the right lower eyelid and midface, accompanied by volume loss, epiphora, and lower‐lid lag. The patient elected for PALF.

Percutaneous aponeurotomy was done to release scar tissue from the patient's prior reconstruction. We harvested adipose tissue from the patient's abdomen, emulsified it, and grafted it into the midface to provide structural volumetric support and reduce the risk of recurrent tethering of the scar. Finer fat preparations were placed in the superficial periorbital tissues to improve contour and soft tissue pliability. The patient tolerated the treatment well and had improved lower eyelid and cheek retraction.

### 3.2 Patient 2

**Figure 1:**
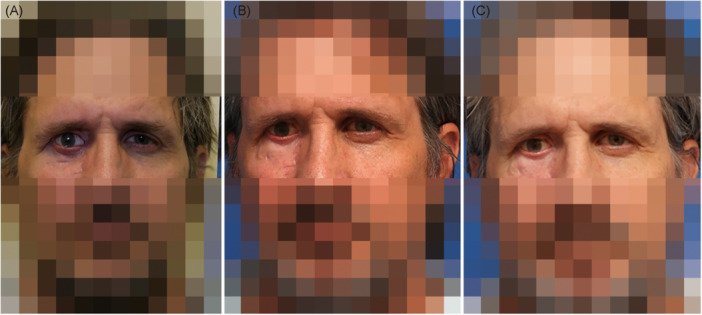
Preoperative views (A and B) and postoperative view (C) of Patient 2, showcasing the improvements 1 year after undergoing PALF. PALF, percutaneous aponeurotomy and lipofilling.

**Figure 2:**
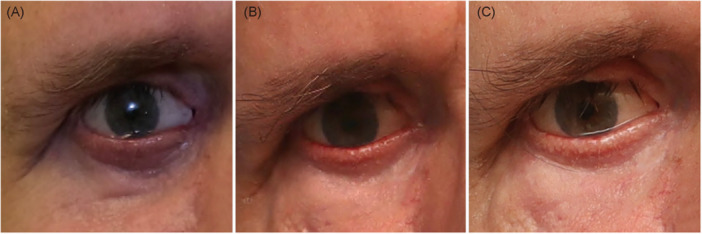
Detailed view of the medial canthus of Patient 2 (A) prior to PALF and (B) one year post‐PALF, illustrating the surgical outcome. PALF, percutaneous aponeurotomy and lipofilling.

Patient 2 presented with facial asymmetry due to right‐sided facial trauma from a workplace injury, causing asymmetric tissue loss in the right periorbital area and lower eyelid retraction (Figures 1A,B and 2A). The volume loss led to recurrent medial canthal detachments and mispositioning of his ocular prosthesis.

**Figure 3:**
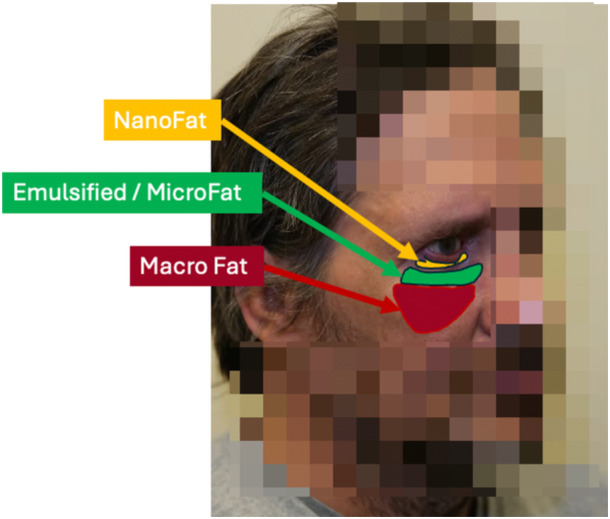
Illustration of nanofat, microfat, and emulsified fat injection sites in Patient 2, demonstrating their roles in providing volumetric support, contour optimization, and ocular prosthesis repositioning.

PALF was performed in the lower periorbital region through two puncture sites, facilitating the release of the lower eyelid retraction. Structural fat grafting was performed for volume restoration, and finer adipose preparations were used in superficial periorbital tissue planes to improve contour and skin quality. We also treated the right cheek area via a pre‐auricular puncture site, where we injected emulsified fat to restore appropriate facial contour (Figure 3).

The volume restoration improved periocular soft‐tissue support, facilitating medial canthal repositioning and enhancing facial symmetry, as well as improving ocular prosthesis positioning. These changes enhanced facial symmetry and contour, resulting in a more balanced periocular appearance and satisfying the patient's esthetic desires (Figures 1C and 2B).

### 3.3 Patient 3

Patient 3 experienced severe facial injury from a gunshot wound, impacting her left zygomaticomaxillary complex and orbit. This caused significant lateral malar depression. PALF was performed twice in the lower periorbital region and left cheek through multiple puncture sites, removing extensive fibrotic scar tethering and alleviating lower eyelid retraction. In this patient's case, this was done in preparation for a suborbital implant. Structural fat grafting was performed to restore malar contour and support the lower lid margin. Finer fat preparations were placed in the superficial tissues to optimize contour, and a suborbital implant was placed once soft‐tissue pliability improved.

## 4 Discussion

Percutaneous aponeurotomy combined with autologous fat grafting demonstrated favorable outcomes in treating periorbital scars in all three patients presented. Notably, each procedure was performed without complications, and each patient experienced significant improvement in symptoms related to their specific periorbital lesion. Based on our experience, this technique may be a useful option for restoring volume, improving soft‐tissue mobility, and enhancing esthetic contour in selected patients. As mentioned above, the principles underlying this approach have previously been described as PALF by Khouri et al. [1], although primarily for the management of burn contractures, radiation fibrosis, and soft tissue deficiencies throughout the body, rather than within the periorbital region. In contrast to large‐scale reconstructive applications, the present series highlights the adaptability of these principles to the periorbital region, where precision, tissue delicacy, and functional considerations are paramount.

Careful patient selection and meticulous release of fibrotic tethering are critical to achieving optimal outcomes. Structural fat grafting following scar release provides volume restoration while helping reduce the risk of recurrent tethering. In select cases, staged treatment may be required, as demonstrated in Patient 3. Further research investigating the long‐term efficacy of PALF is necessary to define better its role in the management of complex periorbital scar deformities.

## Author Contributions

**Daniel Karasik:** writing – original draft, visualization, project administration, formal analysis, investigation. **Alexandra F. Welschmeyer:** writing – review and editing. **Eve Tranchito:** writing – review and editing. **Humzah Quereshy:** writing – review and editing. **Saikrishna Gourishetti:** writing – review and editing. **Cyrus C. Rabbani:** conceptualization, supervision, writing – review and editing, project administration.

## Funding

The authors have nothing to report.

## Ethics Statement

This study was reviewed by the University Hospitals IRB and determined to be exempt (IRB Number: STUDY20231501).

## Conflicts of Interest

The authors declare no conflicts of interest.

## Data Availability

Data sharing not applicable to this article as no datasets were generated or analyzed during the current study.
